# Measurements of Normal Incidence Reflection Loss as a Function of Temperature at the Water-Castor Oil Interface †

**DOI:** 10.3390/s19153289

**Published:** 2019-07-26

**Authors:** Dong-Gyun Han, Him-Chan Seo, Sungho Cho, Jee Woong Choi

**Affiliations:** 1 Department of Marine Science & Convergence Engineering, Hanyang University ERICA, Ansan 15588, Korea; 2 Department of Marine Security and Safety Research Center, Korea Institute of Ocean Science and Technology, Busan 49111, Korea

**Keywords:** acoustic measurements, reflection loss, temperature of intromission, acoustic impedance, water-castor oil interface

## Abstract

Reflection loss at the water-castor oil interface as a function of temperature was measured in a direction normal to the interface using a 200-kHz acoustic signal. The acoustic impedance of water increases with temperature, whereas that of castor oil decreases. The measured reflection losses varied from 30 to 65 dB, and a sharp rising peak in reflection loss was observed at the temperature at which the acoustic impedance of water became equal to that of castor oil. This temperature is called the temperature of intromission in this paper. These measurements were compared with the model predictions based on a Rayleigh-reflection model using the measured sound speeds of both fluids. The sound speeds in water and castor oil as functions of temperature are the input parameters of the Rayleigh-reflection model, and were measured directly using an arrival time difference method in the temperature range of 5 to 30 °C. The comparison results imply that temperature is an important factor affecting the reflection at the interface separating the two fluids.

## 1. Introduction

Acoustic waves propagating in a medium interact with the boundaries between media with different physical properties. If the roughness of the boundary is negligible, the acoustic interactions are the reflection and transmission at the boundary. Numerous efforts have been made to understand the phenomena of acoustic interactions at the boundaries between two media with different characteristic impedances [1,2,3,4], and such results have been applied to various areas of acoustics, including architectural acoustics [5], bioacoustics [6], ultrasonics [7,8], and underwater acoustics [9,10,11,12,13,14,15,16,17,18,19,20,21,22,23].

One representative but simple boundary is a fluid-fluid boundary, which can be explained using a Rayleigh reflection and transmission model [10,11]. Most studies analyze the acoustic interaction at the boundary as a function of the grazing angle [9,12,13,14,15]. For example, if the sound speed in the second medium is higher than in the first, reflection loss increases with the grazing angle and a critical angle exists. Transmission into the second medium does not occur at grazing angles less than the critical angle. On the other hand, if the sound speed in the second medium is lower than in the first, total transmission into the second medium occurs at a specific angle, which is referred to as the angle of intromission.

These theoretical results have been verified experimentally [16,17,18], and acoustic parameters for both media (e.g., sound speeds, densities, and attenuations) have been inversely estimated through comparison with the model predictions [19,20,21,22]. However, few measurements have assessed reflection from the boundary as a function of temperature, and the effect of the temperature on the acoustic interaction at the boundary has been overlooked.

In this paper, we investigate reflection loss as a function of the temperature instead of the grazing angle. Reflection and transmission at the boundary depend on the characteristic acoustic impedances of the two media. The impedance of a plane wave is the product of the sound speed and the density of the medium [23]. Because the sound speed and density vary with temperature, variations in the reflection loss can also occur. In this paper, two fluids, water and castor oil, were considered. Castor oil has been used in underwater acoustics as a sound transfer medium between sonar elements and water, because the acoustic impedance of castor oil is similar to that of water [24,25,26]. However, the acoustic impedance properties of water and castor oil for temperature are different [27,28,29]. For example, the acoustic impedance of water increases with temperature, while the impedance of castor oil decreases. Reflection loss at the water-castor oil interface was measured in the laboratory and a sharp peak in reflection loss was observed at a specific temperature, which is hereafter referred to as the *temperature of intromission*. Because castor oil’s nominal density of 950 kg/m^3^ is lower than that of water, the pale yellow liquid floats on water [23,30]. For this reason, acoustic measurements were made using a transducer installed on the bottom in a small tank, looking upward. The paper is organized as follows. A description of acoustic measurements taken in this study, including the sound-speed measurements in the two fluids and reflection-loss measurements as a function of temperature at the water-castor oil interface, are given in Section 2. Section 3 outlines the reflection-loss model based on a two-layered homogeneous fluid structure, in which the temperature of intromission is introduced. The measurements are presented and compared with model predictions in Section 4. A summary and discussion follow in Section 5.

## 2. Acoustic Measurements

### 2.1. Sound-Speed Measurements

**Figure 1:**
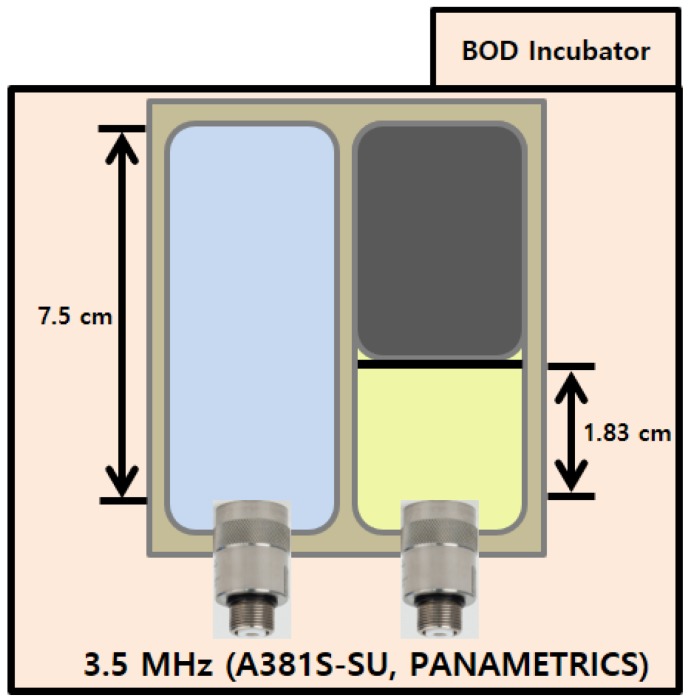
Acoustic measurement system to measure the sound speeds in water and castor oil (top view).

Reflection loss varies depending on the sound speeds and densities of the upper and lower media. Thus, it is important to precisely estimate the values of these parameters. In general, the sound speed varies with temperature of the medium and accordingly the reflection coefficient also varies with temperature. The sound speeds in water and castor oil as a function of temperature were directly measured using monostatic ultrasonic transducers with a center frequency of 3.5 MHz (A381S-SU, PANAMETRICS) installed at one end of a Teflon container (Figure 1). The Teflon container has two rooms: one with a length of 7.5 cm was filled with water and the other, with the length of 1.83 cm, was filled with castor oil. A 0.5 µs continuous wave was transmitted, and an echo signal reflected from the other end of the container was received by a transducer. Because the attenuation of castor oil is much higher than that of water, no echo signal was observed in castor oil when using a container of the same length as the one used for the sound-speed measurements in water. For example, the attenuation in castor oil at a frequency of 3.5 MHz is about 7.3 dB/cm at 20 °C [31], whereas that in fresh water is about 0.02 dB/cm at 20 °C [32]. The sound speed can be estimated by dividing twice a room’s length by the arrival time. Because the error in sound-speed measurements decreases as the distance of propagation increases, longer containers were used to measure the sound speed in water.

**Figure 2:**
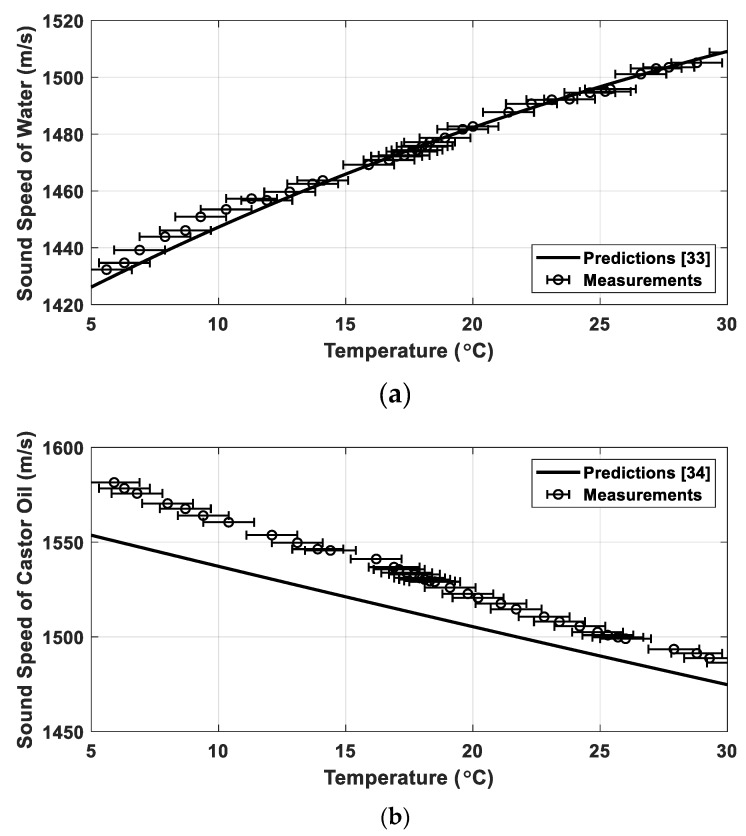
(**a**) Measured sound speeds in water (circles) as a function of temperature and a comparison with the predictions obtained by an empirical formula (solid line) and (**b**) those for castor oil.

The sound-speed measurements of water and castor oil as functions of temperature were made for 36 and 37 different temperatures, respectively, ranging from 5 to 30 °C (Figure 2). For each temperature step, acoustic transmission was repeated 30 times and the mean sound speeds for both media were calculated. The standard deviations of sound speed for both media were estimated to be less than 1.3 m/s for each case. The measured sound speeds were compared with model predictions obtained using an empirical formula found in references [33,34]. Horizontal error bars in the measurements indicate sound-speed uncertainties estimated with a thermometer accuracy of ±1 °C. The measured sound speeds in water were in close agreement with the modeling outputs, whereas the measured values in castor oil were 10–30 m/s higher than predicted. The sound speed in water increases with temperature while it decreases in castor oil. Approximately 90% of the castor oil is triglyceride formed from ricinoleic acid (C_18_H_34_O_3_) [30]. However, the chemical composition of the castor oil may vary with the process of extraction, filtration, and refining, potentially causing sound-speed mismatch between the measurements and model predictions. Sound speed c is determined by density ρ and bulk modulus K, which is given by [35]:

(1) $$c=\sqrt{\frac{K}{\rho }}$$

In the case of castor oil, bulk modulus and density both decrease as the temperature increases. However, the decrease of the bulk modulus is greater than that of the density and thus the sound speed decreases. On the other hand, in the case of water, the bulk modulus increases with the temperature at temperatures below ~50 °C and decreases at temperatures higher than ~50 °C [36]. The density of water decreases with the temperature. Accordingly, the sound speed in water increases with temperatures up to ~70 °C and then decreases with the temperature [37].

### 2.2. Reflection-Loss Measurements

**Figure 3:**
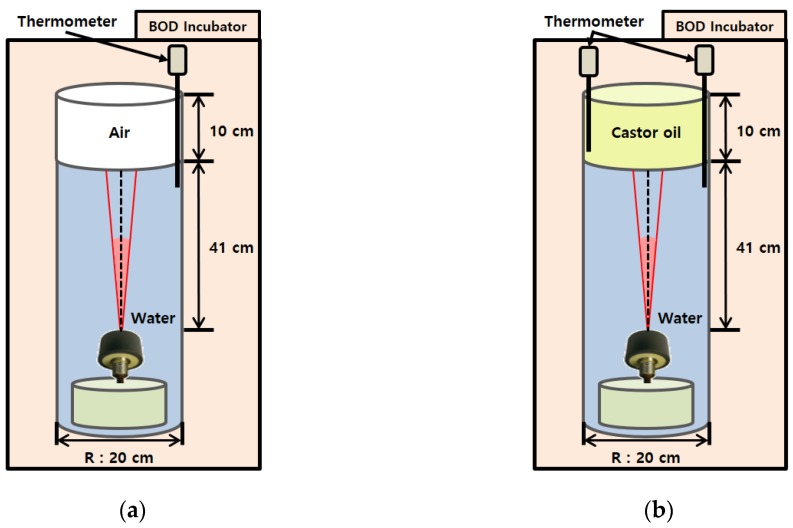
Acoustic measurement system for reflection loss as a function of temperature for (**a**) a water-air interface and (**b**) a water-castor oil interface. The near-field transmission-loss measurements in the water medium were first made in the case of water-air interface, and the results were applied to reflection-loss measurement for a water-castor oil interface.

Measurements of reflection-loss as a function of temperature were made in a cylindrical glass tank with an inner radius of 10 cm and a height of 86 cm, which was installed in a low-temperature biochemical oxygen demand (BOD) incubator (VS-3250Bi-L, VISION SCIENTIFIC Co., Ltd., Daejeon, Korea) to control the temperatures of water and castor oil (Figure 3). A directional transducer (200-7G, SIMRAD) with a main lobe beam width of 7° was placed on the tank bottom looking upward. A continuous wave (CW) with a center frequency of 200 kHz and a pulse length of 0.025 ms was transmitted as a source signal, and the echo was recorded at a sampling rate of 2.5 MHz. The reflection loss RL can be expressed by a sonar equation given by [23]:

(2) RL=SL−2TL−SPL

where SL is a source level (dB re 1 μPa at 1 m), TL is transmission loss (in decibels, dB) from the source to interface, and SPL is a sound pressure level of the received signal (dB re 1 μPa). TL can be predicted by 20logr (where r is the propagation range) under the assumption that the sound propagates spherically in the far-field region. The critical distance between the near-field and the far-field is defined as $\pi a^{2}/\lambda$, where a and λ are the radius of the radiating face area of the transducer and the acoustic wavelength, respectively [38,39,40]. The critical distance was estimated to be ~4 m and the TL may not exhibit a spherical spreading loss. SL−2TL can be estimated directly from the echo signal reflected from the water-air interface, which can be considered an impenetrable boundary with a reflection coefficient of −1 [10].

The measurement of SL−2TL was made in the water tank filled with water only, as shown in Figure 3a. Because the distance between the transducer and the water-air interface is 0.41 m and the beam width of the main lobe is 7°, the radius of the area insonified dominantly at the interface was estimated to be less than 3 cm, which is significantly smaller than the radius (10 cm) of the water-air interface of the tank. A thermometer (YT-9201, UINS, Seoul, Korea) was placed in the tank to monitor temperature variation. A source signal was transmitted 10 times and the intensity averaged. This process was repeated 9 times within a water temperature range of 6.7 to 30.3 °C. After measuring SL−2TL, the tank was filled with 10-cm-thick castor oil (Figure 3b). Another thermometer monitored the temperature of the castor oil. Acoustic measurements were repeated 34 times within a temperature range of 7.1 to 29.8 °C, when the temperatures of both fluids were equalized after sufficient time was allowed in the incubator.

## 3. Reflection-Loss Predictions

**Figure 4:**
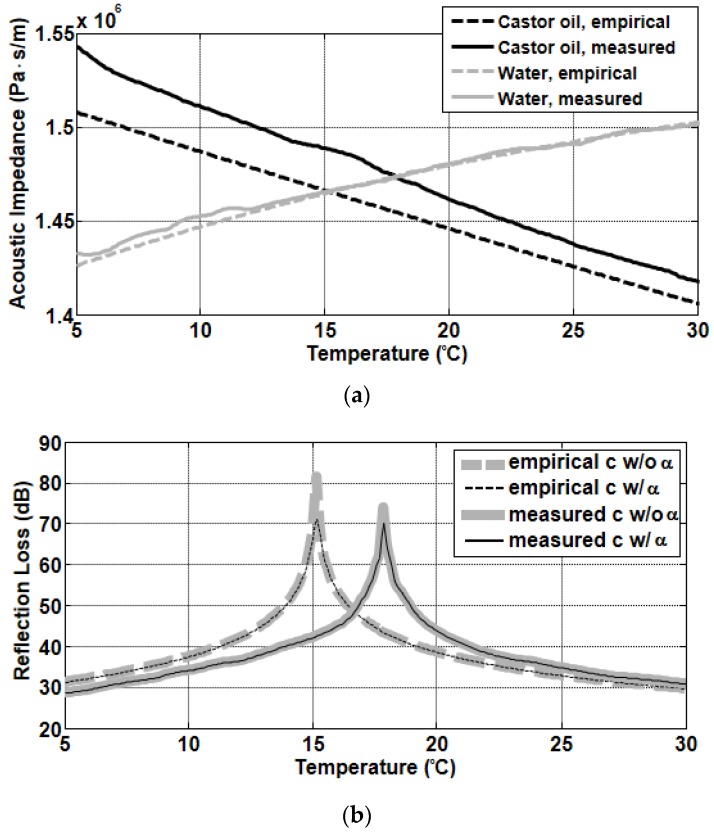
(**a**) Acoustic impedances for normal incidence of castor oil (black lines) and water (gray lines). (**b**) Reflection-loss predictions for water-castor oil interface obtained using $-20\log_{10}\left|R\right|$ based on the Rayleigh reflection coefficients using acoustic impedances in (**a**). Thin and thick lines in (**b**) represent the reflection-loss predictions with and without the castor oil’s attenuation, respectively. In both (**a**) and (**b**), solid and dashed lines indicate the predictions estimated using the sound speeds measured and predicted by the empirical formula, respectively.

A Rayleigh reflection coefficient R for the sound incident in a direction normal to the interface is calculated by [10,12,41]:

(3) $$R=\frac{\rho_{c}c_{c}-\rho_{w}c_{w}}{\rho_{c}c_{c}+\rho_{w}c_{w}}=\frac{\rho_{c}k_{w}-\rho_{w}k_{c}}{\rho_{c}k_{w}+\rho_{w}k_{c}}$$

where k is the acoustic wavenumber. Physical parameters with subscripts c and w indicate the parameters corresponding to castor oil and water. The product of density and sound speed is called acoustic impedance for normal incidence [10]. The attenuation in the castor oil can be considered by assuming the wavenumber in castor oil to be complex as below [20,42]:

(4) $$k_{c}=\frac{\omega }{c_{c}}\left(1+i\frac{\alpha_{c}}{40\pi \log e}\right)$$

where, ω and $\alpha_{c}$ are the radial frequency and the castor-oil’s attenuation in dB/λ, respectively. Reflection loss is defined by $-20\log_{10}\left|R\right|$ and expressed in dB. Figure 4a shows the acoustic impedances for normal incidence obtained using the sound speeds measured in this study and predicted by the empirical formula and the densities from references [34,43]. The densities of castor oil and water have been reported to vary with temperature in the range of 953–970 kg/m^3^ [34] and 995–1000 kg/m^3^ [43], respectively. Although efforts were made to measure the densities of both fluids, measurement errors were significantly higher than the ranges mentioned above. Instead, the densities suggested in the references were used. In the case of castor oil, the acoustic impedances obtained using a measured sound speed fell from ~1.54 × 10^6^ Pa·s/m at 5 °C to ~1.42 × 10^6^ Pa·s/m at 30 °C, and those using predicted sound speed fell from ~1.51 × 10^6^ Pa·s/m at 5 °C to ~1.41 × 10^6^ Pa·s/m at 30 °C. In contrast, the acoustic impedances in water for both cases increased from ~1.43 × 10^6^ Pa·s/m at 5 °C to ~1.50 × 10^6^ Pa·s/m at 30 °C. Figure 4b shows the model predictions for $-20\log_{10}\left|R\right|$ based on Equation (3) using the acoustic impedances shown in Figure 4a. The predicted values increased rapidly with temperature up to 15.2 °C and 17.9 °C when sound speeds predicted by the empirical formula and the measured sound speeds are used, respectively, and then decreased above these temperatures. The temperature corresponding to the peak in the reflection-loss prediction is the temperature of intromission, which means the acoustic energy incident to the interface at this temperature is transmitted in total to the second medium without upward reflection. Note that the temperature of intromission occurs at a cross point between the acoustic impedance lines of the upper and lower media, shown in Figure 4a, which are 15.2 °C and 17.9 °C, respectively, for the case that the predicted and measured sound speeds are used. At 200 kHz, the attenuation of sound in castor oil decreases with temperature from 0.049 dB/cm at 5 °C to 0.022 dB/cm at 30 °C [44], and in this case, the attenuation of castor oil is only sensitive near the temperature of intromission, reducing the peak level as shown in Figure 4b.

## 4. Results

**Figure 5:**
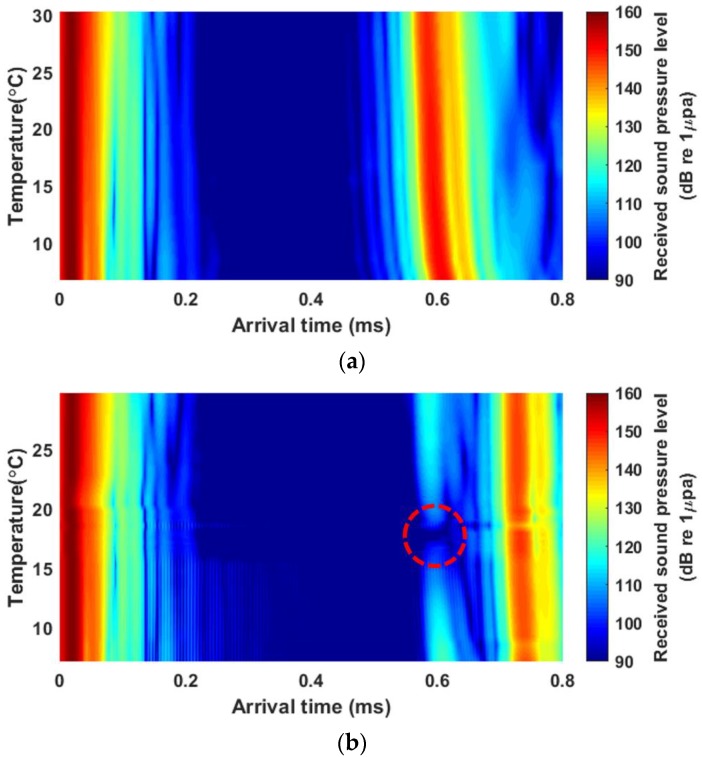
Received sound pressure levels as functions of temperature and arrival time for (**a**) a water-air interface and (**b**) a water-castor oil interface. The average intensities of 10 pings for each temperature step were interpolated. For the water-air interface and the water-castor oil interface, nine and thirty-four datasets, respectively, were used for the interpolation. The red circle in (**b**) highlights where the total transmission into the castor oil layer occurred. The temperature of this part corresponds to the temperature of intromission.

**Figure 6:**
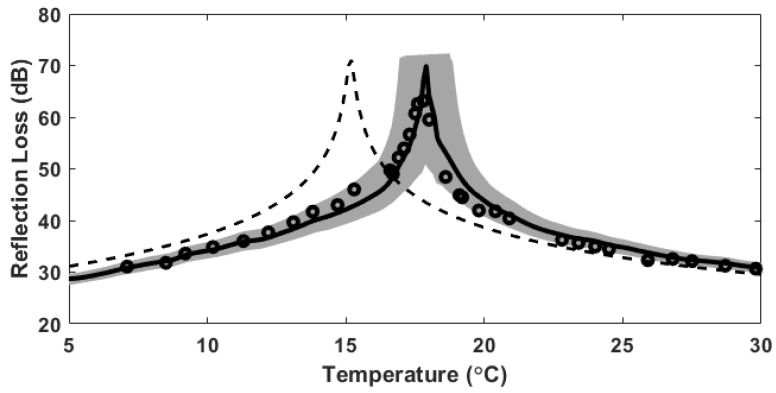
Reflection-loss estimates (circles) as a function of temperature for the water-castor oil interface and the comparison with model predictions obtained by the Rayleigh reflection model. The solid and dashed lines represent the model curves predicted using the sound speeds measured and estimated by the empirical formula, respectively. The gray shaded area is the error range of reflection loss associated with the uncertainty of the sound speed due to thermometer accuracy.

Figure 5 shows the arrival structures of the received sound pressure levels versus temperature. In a case of the water-air interface (Figure 5a), dominant signals reflected from the interface arrived at approximately 0.6 ms, and the energies were relatively high owing to the total reflection at the water-air interface. In addition, there was a tendency for the arrival time to decrease slightly as the temperature increased because the sound speed increased with temperature. In the case of the water-castor oil interface (Figure 5b), the signals reflected from the interface arrived at approximately 0.6 ms and were followed by signals with much higher energies reflected from the castor oil-air interface after an interval of approximately 0.13 ms. Since a 0.025 ms long CW-pulse signal was transmitted, the signals reflected from two interfaces were time resolved. Note that the signals reflected at the water-castor oil interface were not observable near 17.9 °C, which is the temperature of intromission, as indicated by the dashed red circle in Figure 5b. Figure 6 shows reflection losses as a function of temperature estimated for the water-castor oil interface and compared with model predictions for $-20\log_{10}\left|R\right|$ based on Equation (3). The reflection-loss measurements agreed relatively well with the model curve predicted using the measured sound speed, whereas they disagreed with the model curve predicted using the sound speed calculated by the empirical formula, especially near the temperature of intromission.

## 5. Summary and Discussion

Measurements of reflection loss as a function of temperature at the water-castor oil interface were made using a 200-kHz CW signal to investigate the effect of temperature variation on reflection loss. The measurements were carried out in a direction normal to the interface (corresponding to the grazing angle of 90°) in the BOD incubator to control the temperatures of two media. The sound speeds as a function of temperature in the two media were directly measured by the arrival time difference method using 3.5 MHz signals, which were used for model predictions for $-20\log_{10}\left|R\right|$ with the Rayleigh reflection coefficient. The measured sound speeds in water showed a tendency to increase with temperature, in close agreement with previous studies. On the other hand, the sound speeds in castor oil decreased as temperature increased and the measurements were somewhat higher than those estimated by the empirical formula. This mismatch seems to arise from the difference in castor oil quality, which depends on the production process.

In the reflection loss data measured as a function of temperature, a sharp rising peak of reflection loss was observed at a specific temperature, referred to here as the temperature of intromission, which for the water-castor oil interface was estimated to be ~17.9 °C. The temperature of intromission corresponded to the temperature at which acoustic impedance of water became equal to that of castor oil obtained using the measured sound speed.

**Figure 7:**
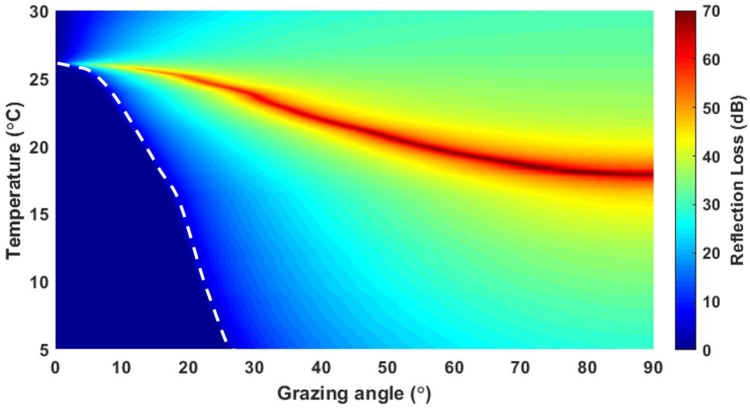
Reflection-loss simulation as functions of grazing angle and temperature for the water-castor oil interface. White dashed line represents the curves corresponding to critical angles, which varies with temperature and grazing angle. Red area corresponds to the area where the total transmissions occur.

Figure 7 shows the reflection loss predictions as functions of grazing angle and temperature for the water-castor oil interface, in which the sound speeds as a function of temperature obtained by an interpolation of the measurement values (shown in Figure 2) and densities predicted by the empirical formula were used. Based on our sound-speed measurements, the sound speed of castor oil was higher than that of water at the temperature less than ~26 °C. Accordingly, below this temperature, the total reflection occurs at a grazing angle less than the critical angle, which varies with temperature as marked by white dashed line. As mentioned earlier, acoustic impedance of water increases with temperature and that of castor oil decreases. The total transmission from water to castor oil occurs at the temperature where the acoustic impedances of both fluids are equalized, which is corresponding to the red area in Figure 7. For these reasons, interestingly, it is observed in the simulation that both total reflection and total intromission at water-castor oil interface exist between about 17.9 °C and 26 °C. For example, in the case of temperature of 22 °C, the total reflection and total intromission are simulated to occur at the grazing angles of 10.2° and 39.7°, respectively.

Our results may be applied in several ways, including the development of underwater acoustic sensors [45,46], the separation of water in oil products in the petroleum industry [47], and acoustic detection of hazardous and noxious substances (HNS) spilled at sea [28]. For example, a piezoelectric transducer is encapsulated for use in underwater environments. There are two main types of encapsulation materials: polyurethane and castor oil. Although there are several reasons to use polyurethane as an encapsulation material, such as its cost and manufacturability, oil-filled transducers are still heavily recommended because they are capable of producing a higher power output than polyurethane transducers [48]. Another example is an acoustic method to monitor the oil-water interface in an oil tank. It is important to separate the oil from water so as to not pump out any oil with the water inside an oil tank, for which the acoustic method can be useful [47]. Last, our results may be applied to the detection of HNS deposited on the seabed after an HNS spill accident [28]. Because it is difficult to directly observe substances that have sunk to the seabed, an acoustic method can be used to detect or monitor such substances. There are many liquids that can form an interface with water. These liquids may have specific gravities similar to that of water, and they usually vary with temperature. When the acoustic impedance of a substance becomes equal to that of water, total transmission can occur, in which the substance is undetectable via acoustic observation. However, there are few studies on the effects of acoustic interactions at the boundary on the temperature variation of fluids. Further study of the acoustic behaviors of substances is still needed.
